# *Tumblr Facts*: Antecedents of Self-Disclosure across Different Social Networking Sites

**DOI:** 10.3390/ejihpe12090087

**Published:** 2022-09-01

**Authors:** Marcella Bianchi, Rosa Fabbricatore, Daniela Caso

**Affiliations:** 1 Department of Humanities, University of Naples Federico II, 80133 Naples, Italy; 2 Department of Social Sciences, University of Naples Federico II, 80138 Naples, Italy

**Keywords:** online self-disclosure, social anxiety, negative emotionality, self-esteem, preference for online social interactions, social compensation, enhancement

## Abstract

Previous research investigating antecedents of online self-disclosure pointed out two perspectives: social compensation and enhancement hypotheses, showing controversial findings. Furthermore, most contributions have focused on social networking sites (SNSs) considered mainstream, like Facebook and Instagram, and such results are often considered universally valid for all SNSs. Tumblr is a less-studied SNS with peculiar features—such as anonymity, higher control over the presentation of personal aspects, supportive communities—that could particularly lead individuals to self-disclose. As prior contributions highlighted that the features and affordances could define how a medium will be used, this paper aims to investigate the antecedents of online self-disclosure on Tumblr and other mainstream SNSs. We run a survey on 559 Tumblr users (aged 13–70; M = 28.86; SD = 12.34). T-test showed that Tumblr users have a higher willingness to self-disclose on Tumblr compared to another SNSs (*t* = 22.44, *p* < 0.001). A path analysis model confirmed the predictive role of some psychological variables on self-disclosure on Tumblr but not on mainstream SNSs. In particular, self-disclosure on Tumblr was predicted by self-esteem, negative emotionality, and preference for online social interactions, which was in turn predicted by social anxiety. These findings partially supported both social compensation and enhancement hypotheses, indicating that the phenomenon is more complex than expected.

## 1. Introduction

Psychosocial literature is increasingly involved in studying social networking sites (SNSs) and their impact on users’ social experience [1,2]. The proliferation of smart devices has been accompanied by the increasing availability of opportunities for online self-disclosure, which is defined as a variety of cyberbehaviors (e.g., instant communication, microblogging) aiming to convey information, maintain communication, or satisfy social needs in cyberspace [3] by sharing personal information with others [4]. Despite the growing interest in studying online self-disclosure, most contributions tend to focus on SNSs considered mainstream, such as Facebook, Instagram, and Twitter [5]. Nonetheless, the literature investigating this phenomenon in other virtual spaces is much sparser and unsatisfactory [6]. As Brailovskaia and Margraf [6] pointed out, most of the earlier studies have been conducted on Facebook, and such results are often considered universally valid and applicable to all SNSs. This bias seems particularly relevant, considering that previous contributions highlighted that the characteristics and affordances of a medium are not background elements, but can define in unexpected ways how it will be used and which users will find it suitable for their intentions [7,8,9]. According to Griffith and Stein [10], each SNS has its own reputation based on branding and self-selected user communities, meaning that the same person could tend to select different SNSs for different uses. That could explain why 98% of users who are active on a social platform are also active at least on one other SNS [11]. In the broad panorama of currently available platforms, Tumblr is a unique SNS for its characteristics, such as total anonymity and a distinctive cultural impact [12]. Recently, by quoting a young interviewee, Keller [13] defined Tumblr as a “Proper Black Hole” (p. 7), as it grants users a sense of freedom and identity protection from the constraints of more traditional networked social media. Coherently, Renninger [8] indicated that the ways users developed platform-specific social norms based on Tumblr’s affordances determined the platform’s popularity, especially (and not only) among marginalized groups that prioritize privacy, community, and respect. However, despite many interesting contributions, few studies focused on Tumblr by adopting a psychological framework and employing quantitative methods [6].

Because of its distinctive qualities and affordances and the lack of related literature, this study aims to investigate differences in the antecedents of online self-disclosure on Tumblr and other mainstream SNSs through quantitative methods.

### 1.1. Tumblr Peculiarities

Tumblr is a hybrid platform characterized by some features typical of SNSs and, in other respects, similar to traditional blogs [7]. It was founded in 2007 by David Carp, and during 2014 was the fastest-growing SNS, with an increase of +120% in active users between the first and the third quarters of the year [14]. However, after being known for the total absence of censorship, which resulted in the proliferation of any kind of content—including pornography—Tumblr banned “adult content” in 2018. This event exacerbated the decline in Tumblr traffic, which was already declining and further diminished from 521 million in December 2017 to 370 million in February 2018 [15]. Currently, Tumblr is the 15th most used social platform in Italy, with 11.5% of users affirming use of it [16].

For the affordances it offers, the sub-communities it grants, and the practices that its users have developed, Tumblr can be considered not entirely comparable to other SNSs [8,16,17,18]. Among its particular affordances [17], anonymity is paramount: on Tumblr, users identify themselves through pseudonyms, and the social network they build there is often entirely independent of their offline contacts [18,19,20,21]. In addition, Tumblr’s relational network implies an explicit distinction between followers and followed [22], giving the user complete freedom to choose the blogs whose new posts will be shown on their *dashboard* [19]. Moreover, whereas on most mainstream SNSs users create a profile that includes personal information such as age, gender, place of affiliation, interests, and educational background, Tumblr does not offer these same possibilities. Instead, the process of structuring the online identity on Tumblr relies on a combination of features and takes place through the creation of blog posts, the *reblog* of various types of content already present in the network, the utterly customizable blog aesthetic (or *theme*), the use of tags, and, in particular, through the *About Me* and *Bio* pages, in which they can freely decide which information to include, without a pre-established format [7,23]. Furthermore, using the tag system—which allows both spaces and punctuation—it is possible to relate directly to the content without necessarily having to interact with other users [7,19,24]. Therefore, Tumblr users are offered peculiar identity experimentation opportunities [17,22,25], which could lead them to engage in disclosing aspects of themselves that would not be easily expressed in conditions of greater recognition, like offline interaction or less anonymous SNSs, such as Facebook [18,21].

Despite the centrality of content rather than direct interaction between users [8], more than other SNSs, Tumblr managed to create a solid internal sense of belonging so that it is possible to speak of a large community with its own uses, ceremonies, and languages [19], also articulated in sub-communities founded on shared experiences, such as gender labels [7,18,21] or interest in a specific topic or fandom [26,27]. Rather than original content creation (the prerogative of only 10% of active users), one of the main functions around which these communities are built is the *reblog* function [8,19,20]. Through this option, a user can quickly insert any type of content found in the network into their blog, with the possibility of adding a comment, an image, or another link, often to highlight some specific aspect or to relate it to others; moreover, every time content is added, a wording automatically appears under it that reveals the source from which it was obtained. This feature is relevant because it makes user participation an integral part of the success of both the content and the platform itself, where not only does participation mean the creation of content but also interaction with it [19,24]. Alongside the *reblog*, the comment, and the *Like*, other possibilities of direct interaction between users are the private chat and the *Ask*. This function allows users to ask other users a question (or leave a message)—anonymously or not. The recipient decides whether to answer: If he chooses to do so, the question and the related answer would appear on his blog like any other post.

According to Yoder et al. [23], Tumblr’s scholarship can roughly be divided into computational research (including network analysis, recommendation systems, and search tools to identify relevant or problematic content) and social–scientific approaches. Among this second line of research, the most-studied topics are mental health, sexuality, and other identity issues—as well as their intersection with media, culture, and fandom [8,10,12,26,28,29,30,31,32,33]. In addition, many studies also emphasized its affordances for marginalized groups—such as LGBTQ+ communities—to explore and construct their own identities, also challenging dominant binary conceptions of gender and sexuality in a context that felt much safer than mainstream and identity-bound media [7,21,31]. For instance, Hawkins and Haimson [31] pointed out that Tumblr may create novel opportunities for trans people to combat widespread violence and stigmatization, possibly enhancing positive mental health benefits in three ways: (1) as a therapeutic outlet to discuss emotions throughout the transition process; (2) as a place to observe others’ transition-related content; and (3) as a way to interact with and positively impact others. Such a finding is consistent with Griffith and Stein [10]. In addition to confirming that posting content on Tumblr is often considered therapeutic on its own accord (regardless of interactions with others), these authors showed that Tumblr communities could be quite active in supporting their members’ mental health disclosures.

Although posting content on Tumblr seems able to provide mental health benefits, some authors [29,34] also suggested a negative perspective, pointing out that connections on Tumblr are often indirect, short-term, and anonymous, and therefore can be toxic. In this regard, referring to queer users, Cavalcante [29] pointed out that the intense emotional investments can lead Tumblr users to *vortextuality* [35], an experience of being dragged into an online black hole.

Anyway, in most cases, the research mentioned above employed qualitative methods like semi-structured interviews [21] and analysis of posts’ content [7,10], focalizing specifically on users from particular communities. To the authors’ knowledge, just a single study employed quantitative methods for investigating psychological variables in the general population [6]. These authors conducted exploratory research showing the correlation between several SNS memberships (including Tumblr), personality traits, and mental health measures. In their study, the use of platforms that focus more on written interaction (namely, Tumblr and Twitter) was negatively associated with positive mental health variables and positively with negative mental health symptoms, including stress and anxiety. However, no study investigated self-disclosure antecedents in a sample of Tumblr users with a quantitative approach.

### 1.2. Online Self-Disclosure and Its Antecedents: Social Compensation vs. Enhancement

The pervasive role of the Internet in contemporary society has led to important changes in the way people communicate about themselves. During the last three decades, scholars have studied the impact of Internet-based technologies on users’ self-disclosure, defined as the process of sharing personal feelings, thoughts, experiences, or information with others [4]. Since its first formulation, this construct has emerged as a crucial element in the relationship development process [36], and more recently many theories and empirical studies have focused on explaining its peculiar processes within online contexts [37,38,39]. Researchers investigating the antecedents of online self-disclosure pointed out two different hypotheses: According to the social compensation hypothesis (also known as “poor-get-richer”) [40,41], individuals who mostly tend to struggle in offline social situations would consider more than others Internet and computer-mediated communication in general as an opportunity to express themselves and engage in social relationships, which would turn into a preference for online social interaction (POSI) over face-to-face communication [42,43] and a higher tendency to self-disclose online. Caplan [42] defined POSI as “a cognitive individual-difference construct characterized by beliefs that one is safer, more effective, more confident, and more comfortable with online interpersonal interactions and relationships than with traditional FtF social activities” (p. 629). Previous studies suggested that POSI is strongly predicted by low self-esteem and high social anxiety [44,45], among the others. Social anxiety is characterized by extreme threat expectancies in social-evaluative contexts, causing avoidance of these situations [46]. Such avoidance often results in subtle safety behaviors, such as speaking quickly or overpreparation [47]. Although these behaviors can temporarily reduce anxiety, events such as social successes and positive feedback can be attributed by the subject to his safety behavior rather than personal abilities. As a result, safety behaviors prevent these individuals from learning their tendency to overestimate the likelihood of receiving a negative assessment and underestimate their social skills [46,48]. Researchers speculated that the textual nature of the Internet and the lack of visual cues during online communications would allow socially anxious people to hide and therefore control the elements of their appearance perceived as causing negative evaluations, like sweating and stuttering [48,49,50]. In line with this hypothesis, online communication can be one of these security behaviors, and several characteristics of the Internet could make it particularly appealing for people facing loneliness, depression, social anxiety, and low self-esteem [42,44,45,51,52,53]. These aspects include greater anonymity, greater control over self-presentation, less perceived social risk (personal costs in case of interaction or relationship failure), and less social responsibility towards others [25,42,54,55,56]. As discussed before, such classical Internet-related characteristics appear to be more prominent in Tumblr than in other mainstream SNSs: Thus, compared to them, Tumblr’s communicative context could be more suitable for such individuals.

The second perspective about online self-disclosure’s antecedents is the enhancement hypothesis (or “rich-get-richer”) [40], postulating that socially competent individuals will consider the Internet an additional venue for social interactions. Empirical evidence showed controversial results, at first confirming that socially anxious individuals turned less often to the Internet but were more prone to believing in the advantages of online communication over face-to-face for disclosing intimate topics [40]. Forest and Wood [57] found that people with low self-esteem considered Facebook an appealing venue for self-disclosure and spent as much (or more) time using it as people with high self-esteem. Still, their disclosures’ low positivity and high negativity elicited undesirable responses from other users, preventing them from reaping its potential social benefits in terms of *Likes* and comments. The authors discuss whether the generally negative disclosures of people with low self-esteem always make them dislikeable or whether such disclosures mainly appear objectionable on Facebook. Hollenbaugh and Ferris [58] found a negative impact of self-esteem on the breadth of self-disclosure on Facebook. Recently, Griffith and Stein [10] examined personal disclosures about mental illness and the responses of online community members among public blog posts of 14,626 Tumblr users, highlighting a significant interaction effect on mental health disclosure frequency and community responses. Although the content of most examined personal disclosures was related to users’ emotions and cognitions about their mental health and feelings of interpersonal loss and change over time, the authors found some differences in online hashtag communities according to the specific level of activity in both posting and commenting and the percentage of question indicators included in the post. Considering these findings, combined with the aforementioned peculiarities of this platform, it is possible to suppose that the online communicative environment created by Tumblr communities is what might prompt users to disclose more about themselves. Thus, it is likely that the communicative environment created by Tumblr communities is what generally prompts users to disclose more about themselves.

Among personality literature, a recent review [1] about how neuroticism (also defined as “negative emotionality”) relates to social media demonstrated that people with high trait neuroticism post fewer and prevalently negative status updates. Negative emotionality refers to an individual’s ability to adjust to their environment [59], with individuals low in this trait typically behaving in “calm” or “stable” ways. In contrast, individuals high in this trait are prone to anxiety, anger, depression, self-consciousness, vulnerability, and impulsiveness [60]. In addition, they appear to react negatively to unpleasant stimuli [61] and to be particularly perceptive to negative experiences [62], concerned with how they appear to others [60], and anxious about their image on social media [63]. In fact, neuroticism is positively related to SNS use for self-promotion [64], sensitivity to rejection, and the need for peer acceptance [65], and it is also associated with a higher tendency to passively use social media [66,67]. According to Bowden-Green et al. [1], current literature about the link between negative emotionality or neuroticism and online self-disclosure is contradictory: For instance, some authors found that neuroticism was positively related to the frequency of social media use, particularly for self-disclosure purposes compared to communication motives [66]; on the contrary, Hollenbaugh and Ferris [58] found that neuroticism negatively impacted the breadth of self-disclosure in Facebook users. Despite these contradictions, the scholarship investigating Tumblr practices showed a general representation of this platform as a safe place to feel protected and freely discuss personal issues [7,18,21,68]. In line with these contributions, Tumblr could be particularly appealing as a virtual place to self-disclose for individuals with high negative emotionality.

### 1.3. The Present Study

The current study aims to investigate differences in online self-disclosure on Tumblr and other mainstream SNSs such as Facebook or Instagram by employing quantitative methods. In particular, we are interested in evaluating the effects of some variables indicated as antecedents of self-disclosures by previous research, namely, self-esteem and social anxiety (both direct and mediated by POSI) and negative emotionality. In this line, we pose the following questions:

RQ1: Will Tumblr users show a higher willingness to self-disclose on Tumblr compared to other SNSs?
RQ2: Will social anxiety, self-esteem, POSI, and negative emotionality directly impact self-disclosure on Tumblr and other SNSs?
RQ3: Will POSI mediate the impact of social anxiety and self-esteem on self-disclosure on Tumblr and other SNSs?

## 2. Materials and Methods

### 2.1. Participants and Procedures

The present study was implemented following receipt of ethical approval by the Department of Humanities of the University of the Naples “Federico II”. Participants were recruited online. A Tumblr account was created at the scope. The blog’s bio introduced the psychological research on Tumblr’s Italian-speaking users and invited participants to join the survey. It was also made explicit that the questionnaire was anonymous, and collected data would have been treated according to privacy norms, only for research purposes. The inclusion criteria were owning a Tumblr account and fluently speaking Italian. As a snowball-sampling technique was employed, users were invited to *reblog* (namely, share it on their blogs) the survey post to make more people know about it. Before filling in the online questionnaire, participants were asked to provide informed consent to join the research. Data included in the analyses were collected in February 2020. The questionnaire required a mandatory answer to each item, so no respondent had missing values.

A total of 559 Italian-speaking Tumblr users participated in this study, aged 13–70 (M = 28.86; SD = 12.34). Most were females (66.9%), 32% were males, and 1.1% affirmed not to identify themselves in binary gender categories. Regarding their sexual orientation, the main part was heterosexual (73.5%), followed by bisexuals (15.2%) and homosexuals (5.4%). Moreover, nearly half of the participants were students (47.4%), with a consistent number of workers (38.4%). Participants’ detailed information is presented in Table 1.

### 2.2. Measures

An online self-report questionnaire was developed according to the study’s aims. It consisted of various sections to assess variables of interest, some questions formulated ad hoc, and a section about participants’ personal information, such as age, gender, sexual orientation, and employment status. The following paragraphs describe different measures in detail.

#### 2.2.1. General Social Networking Site Use

To assess participants’ general use of SNSs, some items were formulated. Firstly, we considered their favorite SNS (“*Among all the social networks sites to which you subscribed, which is the one you spend most of the time on?*”), and other SNSs they regularly use (“*Besides Tumblr, which other social networks do you use regularly?*”). Then we posed some more questions specifically about Tumblr use, collecting data about the period of use (“*How long have you used Tumblr?*”), daily time of use (“*How much time do you spend per day on Tumblr?*”), and interaction with other Tumblr users (“*Do you regularly interact with other Tumblr users (through comments, ask, or private chat)?*”). Moreover, we included 12 items on a 5-point Likert scale (from 0 = “*Never*” to 4 = “*Very often*”) to collect information about the frequencies of some of the most diffuse Tumblr practices.

#### 2.2.2. Preference for Online Social Interactions

The POSI subscale from the Generalized Problematic Internet Use Scale—2 (GPIUS—2) [43] was included in its Italian validation [69]. It is a 3-item self-report Likert scale (from 1 = “Definitely disagree” to 8 = “Definitely agree”), asking participants to indicate their agreement to three affirmations (e.g., “*I prefer social interaction on the Internet to face-to-face communication*”; α = 0.89).

#### 2.2.3. Negative Emotionality

We administered three items adapted from the Big Five Inventory–2—XS (BFI-2-XS) [70]. This 3-item scale evaluates participants’ personality trait of negative emotionality, mainly catching the sub-facets of anxiety (“*I am someone who worries a lot*”), depression (“*I am someone who tends to feel depressed, blue.*”), and emotional volatility (“*I am someone who is emotionally stable, not easily upset,*” reversed). Participants are invited to express their agreement on a 5-point Likert scale (from 1 = “Strongly disagree” to 5 = “Strongly agree”). The reliability of the scale in our study (α = 0.70) did not differ much from the original validation (α = 0.73).

#### 2.2.4. Self-Esteem

The Rosenberg Self-Esteem Scale [71] was administered in its Italian validation [72]. It consists of 10 items (α = 0.92) on a 4-point Likert scale (from 1 = “Strongly disagree” to 4 = “Strongly agree”). High final scores correspond to higher self-esteem.

#### 2.2.5. Social Anxiety

The Italian validation [73] of the Social Interaction Anxiety Scale (SIAS) [74] evaluated the fear experienced within social interactions in general (e.g., “*In social situations I usually feel uncomfortable*”). It consists of 19 self-report items on a 5-point Likert scale (from 0 = “Not at all” to 4 = “Very much”; α = 0.94).

#### 2.2.6. Self-Disclosure on Tumblr and Other Social Networking Sites

Participants’ self-disclosure was assessed using the Self-disclosure Index [75], purposely translated into Italian. It consists of 10 items on a 5-point Likert scale (from 0 = “Not at all” to 4 = “I discuss it fully and completely”) aimed at recording users’ tendency to disclose various aspects of themselves. For each item, participants were asked how much they felt willing to share personal information (e.g., “*How willing are you to disclose by sharing personal information about your deepest feelings?”*). This scale was submitted to each participant twice: Firstly, everyone filled it out thinking about Tumblr. Subsequently, participants with a Facebook profile filled it out once more, referring to that profile. Instead, participants without Facebook were asked to indicate another SNS they used and then filled out the scale thinking about that profile. This scale’s reliability in our study was high (α = 0.91 for the self-disclosure on Tumblr and α = 0.89 on the second SNS).

### 2.3. Statistical Analysis

The statistical analyses were carried out using the R statistical software. The scales’ internal consistency was verified by computing Cronbach’s alpha, and each scale’s scoring was assessed considering the average of single items’ scores after having reversed those items that requested it. To evaluate the associations between the considered variables, Pearson’s correlation coefficients R were calculated. A t-test of the difference between averages was conducted to explore the question of a greater tendency to self-disclose on Tumblr rather than on other SNSs (RQ1).

Moreover, to investigate the other research questions (RQ2, RQ3), a path analysis based on maximum likelihood estimation was carried out using the R package lavaan (Rosseel, 2012). The bootstrapping method was used to test the statistical significance of the indirect effects. Several fit indices were considered to evaluate to what extent observed data supported the hypothesized model. Specifically, the Comparative Fit Index (CFI; Bentler, 1990) and the Tucker and Lewis Index (TLI) [76] were calculated, for which values greater than 0.90 are considered indicative of a good fit [77]. Finally, the Standardized Root Mean Square Residual (SRMR) and the Root Mean Square Error of Approximation (RMSEA) were computed, for which values lower than 0.05 are indicative of a good adaptation [78].

## 3. Results

### 3.1. Tumblr Use: Frequency and Practices

The majority of participants (63%) stated that Tumblr is their favorite SNS, followed by Instagram (24.3%) and Facebook (8.1%). There were mostly longtime users in the sample: 42.9% declared that they had used Tumblr for 5 years or more, 24.2% for 3 to 5 years, 21.3% for 1 to 3 years, and 11.7% for 1 year or less. Regarding time spent per day using Tumblr, 31.5% spent 30 min or less, 30.1% spent between 31 and 60 min, 19.6% spent 1 to 2 h, and 18.8% spent 2 h or more. About practices, as Table 2 shows, the most preferred ones were *reblogging* visual content (M = 3.8; SD = 1.3); sharing/collecting quotes or aphorisms (M = 3.3; SD = 1.3); writing text posts in the form of a personal diary, stories, etc. (M = 3.2; SD = 1.5); and sharing/posting content regarding one or more fandoms or a specific topic (M = 3.0; SD =1 0.3). On the other hand, advertising brands or work was the least preferred practice (M = 1.2; SD = 0.7). Moreover, 57% of the sample stated that they have regularly interacted with other Tumblr users (12.3% in an anonymous form, 44.7% in a non-anonymous form), whereas 43% did not interact directly with others.

### 3.2. Psychological Variables Related to Tumblr Use: A First Look

Descriptive analyses and Pearson correlations among variables are presented in Table 3. Results showed that the preference for online social interaction was negatively correlated with self-esteem and positively associated with social anxiety and negative emotionality. Regarding self-disclosure, the results displayed a positive correlation between self-disclosure on Tumblr and both negative emotionality and preference for online social interaction. Self-disclosure on the second social network positively correlated with self-esteem and negatively correlated with social anxiety and negative emotionality. Moreover, self-disclosure on Tumblr was associated with self-disclosure on the second social network. Concerning self-disclosure on an SNS different from Tumblr, 415 participants (74.2%) affirmed being registered on Facebook and consequently referred to it; 55 participants (9.8%) claimed not to use other SNSs, being excluded from the second administration; 60 participants (10.7%) answered about Instagram; and the remaining part (5.2%) responded about other SNSs. Furthermore, *t*-test results (t = 22.44, *p* < 0.001) revealed a higher willingness to self-disclose on Tumblr than on other SNSs (RQ1).

### 3.3. Results of the Path Analysis

**Figure 1:**
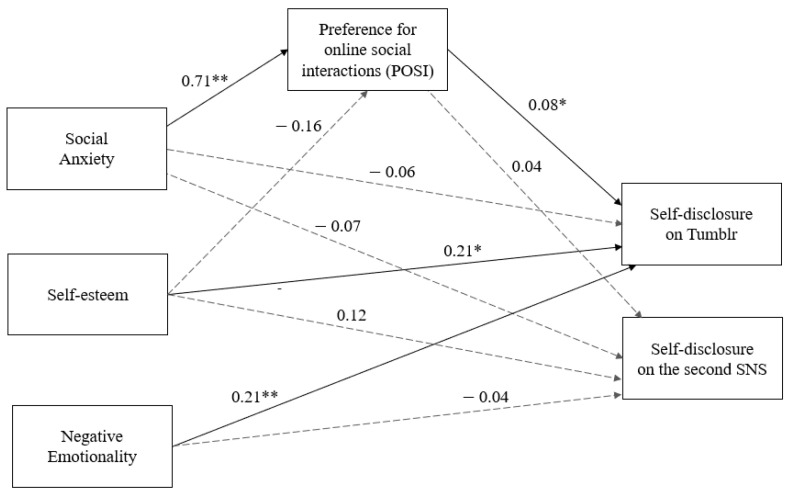
Path model with standardized regression coefficients. Note: The dashed lines indicate not significant paths. ** *p* < 0.01, * *p* < 0.05.

The model estimated to investigate our research questions about Tumblr users’ self-disclosure provided a good fit to the data. All fit indices pointed to a good fit of the global model: χ^2^ = 1.225 (*p* = 0.268), CFI = 0.999, TLI = 0.987, RMSEA = 0.021, SRMR = 0.007. As can be seen in Figure 1, self-disclosure on Tumblr was significantly and positively affected by self-esteem (β = 0.21; *p* < 0.05), negative emotionality (β = 0.21; *p* < 0.01), and preference for online social interactions (β = 0.08; *p* < 0.05). In contrast, social anxiety having no direct effect on self-disclosure was strongly and positively related to the preference for online social interactions (β = 0.71; *p* < 0.01). The latter also fully mediated the impact of social anxiety on self-disclosure on Tumblr (indirect effect: β = 0.05; 95% bootstrapped CI = 0.01 to 0.10). On the other hand, no significant effects were found for self-disclosure on the second SNS, bringing out a difference between the factors underlying self-disclosure on Tumblr and those affecting self-disclosure on another SNS.

## 4. Discussion

As a remarkably increasing phenomenon, the use of SNSs needs to be better explained by psychosocial literature to adequately understand how users could be affected and possibly change their behavior according to the peculiarities of distinct online communicative environments. The current work contributed to this effort specifically by identifying possible differences in antecedents of users’ self-disclosure among mainstream SNSs and less-studied platforms, like Tumblr. Such a goal appears to be valuable since better knowledge of how people refer to SNSs is an essential key to both redesigning such virtual places to be more suitable for different kinds of people and figuring out whether and how these tools could become an opportunity for individuals who are not successful in offline social interactions to express themselves and fulfill their social needs.

Firstly, we found a greater tendency for Tumblr users to prefer that network over other SNSs (Facebook for 74.2% of the sample) as a virtual place for self-disclosing (RQ1). This evidence can easily be interpreted in light of Tumblr’s distinctive qualities—such as anonymity, higher control over the presentation of personal aspects, and participative communities—that create a social environment often alternative to offline networks, so users can reveal hidden parts of themselves, avoiding any risks to their reputation [21,79]. On the other hand, it is also important to consider that the sample’s characteristics could explain such evidence. Notably, we found our participants to mostly be longtime Tumblr users (42.9% of them had used it for more than five years, and 24.2% for 3 to 5 years), and in many cases they identified Tumblr as the SNS they spent the most time on (62.6%), possibly leading them to prefer it for their online disclosure. This interpretation would be coherent with the large body of research supporting the Uses and Gratification Theory [80], which postulates that people choose to use a particular medium according to how well it meets their needs. Therefore, according to this perspective, people who are most active on Tumblr would be so because they get something out of that experience: The co-occurrence of both the preference to disclose on Tumblr and the preference for Tumblr in general as the go-to SNS could suggest that self-disclosure is one of the needs for which they are seeking and finding gratification. Nevertheless, we still cannot firmly exclude the possibility that Tumblr users could also employ it for different motivations, e.g., for adjusting to social pressures [81]. Thus, future research could focus on empirically testing these speculations, for instance, by comparing samples balanced for years of usage and preference, or exploring the relationship between Tumblr vs. other media use and needs met [82].

Further deductions can be drawn from the analysis of paths between psychological variables (RQ2-3). About the direct and mediated impact of considered antecedents on self-disclosure on the second SNS, we identified no significant antecedent, which is inconsistent with previous studies focusing on mainstream platforms [57,58]. By the way, this inconsistency could be attributed to the aforementioned features of our sample, too, which could have resulted in a particular attachment or sense of belonging, leading long-term Tumblr users to not be very interested in disclosing on other SNSs. Although we purposely decided to run our survey on Tumblr users to understand the phenomenon better, future research could investigate such variables on a larger sample, including users from different platforms.

Still, we found a weak positive effect of self-esteem, POSI, and negative emotionality (but not social anxiety) on willingness to disclose on Tumblr. Furthermore, POSI emerged as a mediator in the impact of social anxiety on self-disclosure, but not for self-esteem. Such findings are quite controversial and worth being discussed. First, the direct positive impact of POSI and negative emotionality is in line with the social compensation hypothesis, expecting less socially competent individuals to refer more than others to the Internet for expressing themselves and engaging in social relationships [40,41]. We found no study that empirically tested the direct predictive impact of POSI on self-disclosure. However, Schouten et al. [83] addressed a similar topic while testing their “Internet–attribute–perception” model, according to which two attributes of computer-mediated communication (namely, reduced nonverbal cues and controllability) are considered responsible for increasing adolescents’ use of instant-messaging applications. In this model, such a relationship would particularly depend on the perceived relevance of those characteristics, which would, in turn, mediate the effect of some personality characteristics—including social anxiety—on online self-disclosure. Considering the possible overlap of POSI with the perceived relevance of the features of computer-mediated communication, we could interpret our results as in line with this perspective. Anyway, both the β- and p-value of the impact of POSI on Tumblr self-disclosure suggest that these considerations are far from conclusive and should cue further investigations on the topic.

Besides, in our model, negative emotionality is the strongest antecedent of Tumblr self-disclosure. Although the literature about negative emotionality and online self-disclosure is controversial, our result can be considered consistent with Marciano et al. [66], who found a positive association of neuroticism with the frequency of general online self-disclosure. On the contrary, Hollenbaugh and Ferris [58] showed an opposite direction in negative emotionality’s (and also self-esteem’s) effect on the breadth of self-disclosure in a sample of Facebook users. Such inconsistency could also be attributed to the platforms’ characteristics, although further investigations are still needed (e.g., using the same measure in a sample of Tumblr and Facebook users).

On the other hand, contrary to the social compensation perspective, we also found no significant direct impact of social anxiety on Tumblr self-disclosure and even a positive impact of self-esteem, which could be considered consistent with the enhancement hypothesis instead [40]. Such a result could be discussed in light of Forest and Wood’s [57] perspective. In their study explicitly focused on Facebook users, the authors put forward the hypothesis that the positive opportunities offered by online self-disclosure were particularly attractive for the psychosocially weakest individuals (with low self-esteem), but that, precisely for them, it was also challenging to take advantage of these benefits, showing that “the way people with low self-esteem use Facebook can prevent them from getting its potential social benefits” (p. 300). Interestingly, the authors discovered that people with low self-esteem perceive Facebook as a safe and attractive place for self-disclosure. However, generally, the content of their disclosures is prevalently negative, and there is a lower rate of positive content compared to individuals with high self-esteem. Consistent with the so-called “positivity norm” of Internet culture [84,85], postulating that positive disclosures are generally more frequent and predict positive feedback and higher social support, these data suggest that Facebook’s communicative context, although perceived as attractive, seems not to be structurally suitable to granting such negative disclosures, as users generally prefer not to interact with them (i.e., by pressing the *Like* button). Compared to our findings and the available literature about Tumblr as a supportive community [10,31], this evidence could shed light on the idea that, unlike what was observed about Facebook, Tumblr’s distinctive features could make it a virtual social context in which stigmatization of negative aspects of users communications does not occur—or, perhaps, occurs to a lesser extent. On the contrary, this kind of content is often welcomed, possibly helping individuals receive social opportunities and benefits.

Considering these speculations, taken together, our mixed findings could indicate that the phenomenon is more complex than expected, and such inconsistency with the previous literature on other SNSs suggests that the topic is worth additional attention. In this regard, Luo and Hancock [86] recently proposed a unified framework synthesizing the social compensation and enhancement hypotheses. The authors suggested a bi-directional relationship between self-disclosure on social media and psychological wellbeing, in which several mechanisms and motivations play a role in influencing the paths, including both self-esteem and anxiety. Future research could join the debate by empirically testing these hypotheses and theories on Tumblr users.

### Limitations and Strengths

As the first study to the authors’ knowledge aimed to investigate self-disclosure and its antecedents on Tumblr users through quantitative methods, this work has several limitations and, at the same time, it opens up several possibilities for future investigations.

Firstly, the study was conducted on a convenience sample of Italian-speaking Tumblr users, which significantly limits the results’ generalization to the entire population. In particular, although it seems that most of the non-visual content tends to be in English, it is not uncommon for non-English-speaking users to interact with it, sometimes also expressing themselves in English. In this regard, future research could explore the topic from a cross-cultural perspective. 

Besides, the sample is heterogeneous for many variables, including age. Therefore, future research should employ samples of different age groups to provide a better picture of the phenomenon. Moreover, the study acknowledges all the e-research limitations, like low control over the participants, the impossibility of establishing their identity, and the decrease in attention and answer quality as questionnaire length grows [87].

Furthermore, some of the scales employed are not sensible enough to catch some of the possible nuances of the constructs: For example, the scale employed to measure self-disclosure is monodimensional, but multidimensional scales also exist [58]. Therefore, we can address future studies to investigate these constructs according to such dimensions, also considering the valence of the disclosure (e.g., positive, negative) [57]. Finally, there are limitations related to the proposed model, specifically regarding the possibility of considering some other variables that could mediate or moderate the relationship between our variables of interest, but also regarding possible consequences of self-disclosure in terms of both positive (e.g., wellbeing, social capital) or negative (e.g., Internet addiction) outcomes.

Despite these limitations, the current study highlights that the paths explaining self-disclosure on different SNSs are still worth further investigation and opens many questions for future research. Along with theoretical implications in the debate addressing social compensation and enhancement hypotheses, this study might also have practical implications, as it prompted a better comprehension of the processes underlying how differently characterized SNSs can facilitate people to communicate about themselves. Since the literature about the use of the Internet and SNSs found associations with both positive and negative outcomes [88], it is crucial to acknowledge the distinction between the problematic aspects and the opportunities that SNSs can offer different kinds of users. In particular, this would be useful to shape online platforms in a more valuable way and could inspire interventions aimed at helping users maximize the benefits of SNS usage while avoiding negative outcomes.

## Figures and Tables

**Table 1:** Participants’ sexual orientation and employment status.

	Percentage
**Sexual orientation**	
Heterosexual	73.5%
Homosexual	5.4%
Bisexual	15.2%
Other	5.5%
**Employment status**	
Student	47.4%
Employee	28.4%
Self-employed	10%
Unemployed	11.6%
Running a household	2%
Retiree	0.5%

**Table 2:** Users’ practices on Tumblr.

Practice	M (SD)
1. Reblogging visual content (photos, gifts, pictures, etc.)	3.8 (1.3)
2. Sharing/collecting quotes or aphorisms	3.3 (1.3)
3. Writing text posts in the form of a personal diary, stories, etc.	3.2 (1.5)
4. Sharing/posting content regarding one or more fandoms (TV series/books/actors etc.) or a specific topic (reading, food, animals, etc.)	3.0 (1.3)
5. Knowing/interacting with other users with similar interests	2.6 (1.1)
6. Publishing one’s own graphic contents (photographs, illustrations cartoons, graphic work, etc.)	2.4 (1.3)
7. Sharing erotic and/or sexual materials	1.9 (1.2)
8. Sharing and/or commenting on news	1.9 (1.0)
9. Collecting content from other social networks	1.9 (1.1)
10. Interacting with friends you know even offline	1.9 (1.2)
11. Sharing your knowledge in a specific knowledge field (scientific dissemination)	1.8 (1.0)
12. Advertising your brand or your work	1.2 (0.7)

**Table 3:** Descriptive analyses and correlations among the variables.

	M (SD)	1	2	3	4	5	6
1 Self-esteem	2.64 (0.7)	1					
2 Social anxiety	1.87 (0.9)	−0.64 **	1				
3 Negative emotionality	3.50 (1.0)	−0.70 **	0.60 **	1			
4 POSI	2.97 (1.5)	−0.37 **	0.50 **	0.31 **	1		
5 Self-disclosure Tumblr	1.62 (1.0)	−0.03	0.03	0.13 **	0.10 *	1	
6 Self-disclosure other SNS	0.58 (0.7)	0.18 **	−0.16 **	−0.17 **	−0.01	0.28 **	1

** *p* < 0.01, * *p* < 0.05.

## Data Availability

Data are available on request from the authors.
